# Pharmacological interventions for persistent postural‐perceptual dizziness (PPPD)

**DOI:** 10.1002/14651858.CD015188.pub2

**Published:** 2023-03-09

**Authors:** Katie E Webster, Natasha A Harrington-Benton, Owen Judd, Diego Kaski, Otto R Maarsingh, Samuel MacKeith, Jaydip Ray, Vincent A Van Vugt, Martin J Burton

**Affiliations:** Cochrane ENT, Nuffield Department of Surgical SciencesUniversity of OxfordOxfordUK; Ménière's SocietyDorkingUK; ENT DepartmentUniversity Hospitals of Derby and Burton NHS Foundation TrustDerbyUK; National Hospital for Neurology and NeurosurgeryLondonUK; Amsterdam UMC, Vrije Universiteit Amsterdam, Department of General PracticeAmsterdam Public Health Research InstituteAmsterdamNetherlands; ENT DepartmentOxford University Hospitals NHS Foundation TrustOxfordUK; University of SheffieldSheffieldUK; Cochrane UKOxfordUK

**Keywords:** Adult, Humans, Chronic Disease, Dizziness, Selective Serotonin Reuptake Inhibitors, Serotonin and Noradrenaline Reuptake Inhibitors

## Abstract

**Background:**

Persistent postural‐perceptual dizziness (PPPD) is a chronic balance disorder, which is characterised by subjective unsteadiness or dizziness that is worse on standing and with visual stimulation. The condition was only recently defined and therefore the prevalence is currently unknown. However, it is likely to include a considerable number of people with chronic balance problems. The symptoms can be debilitating and have a profound impact on quality of life. At present, little is known about the optimal way to treat this condition. A variety of medications may be used, as well as other treatments, such as vestibular rehabilitation.

**Objectives:**

To evaluate the benefits and harms of pharmacological interventions for persistent postural‐perceptual dizziness (PPPD).

**Search methods:**

The Cochrane ENT Information Specialist searched the Cochrane ENT Register; Central Register of Controlled Trials (CENTRAL); Ovid MEDLINE*;* Ovid Embase; Web of Science; ClinicalTrials.gov; ICTRP and additional sources for published and unpublished trials. The date of the search was 21 November 2022.

**Selection criteria:**

We included randomised controlled trials (RCTs) and quasi‐RCTs in adults with PPPD, which compared selective serotonin reuptake inhibitors (SSRIs) or serotonin and norepinephrine reuptake inhibitors (SNRIs) with either placebo or no treatment. We excluded studies that did not use the Bárány Society criteria to diagnose PPPD and studies that followed up participants for less than three months.

**Data collection and analysis:**

We used standard Cochrane methods. Our primary outcomes were: 1) improvement in vestibular symptoms (assessed as a dichotomous outcome ‐ improved or not improved), 2) change in vestibular symptoms (assessed as a continuous outcome, with a score on a numerical scale) and 3) serious adverse events. Our secondary outcomes were: 4) disease‐specific health‐related quality of life, 5) generic health‐related quality of life and 6) other adverse effects. We considered outcomes reported at three time points: 3 to < 6 months, 6 to ≤ 12 months and > 12 months. We planned to use GRADE to assess the certainty of evidence for each outcome.

**Main results:**

We identified no studies that met our inclusion criteria.

**Authors' conclusions:**

At present, there is no evidence from placebo‐controlled randomised trials regarding pharmacological treatments ‐ specifically SSRIs and SNRIs ‐ for PPPD. Consequently, there is great uncertainty over the use of these treatments for this condition. Further work is needed to establish whether any treatments are effective at improving the symptoms of PPPD, and whether their use is associated with any adverse effects.

## Plain language summary

**What are the benefits and risks of different medications used to treat persistent postural‐perceptual dizziness (PPPD)?**

**Key messages**

We did not identify any studies that assessed the use of medication to treat persistent postural‐perceptual dizziness (PPPD).

Further work is needed in this area to help establish whether there are any treatments that may be effective at treating this condition, and to check if they cause any unwanted or harmful effects.

**What is PPPD?**

PPPD stands for persistent postural‐perceptual dizziness. People with this condition have symptoms of dizziness or unsteadiness, which are worse when standing up or moving around, or with rich visual stimulation (from complicated patterns or busy moving images).

**How is PPPD treated?**

Sometimes people may take medication to try and help their symptoms of PPPD. This includes medicines known as 'selective serotonin reuptake inhibitors' (SSRIs) and 'serotonin and norepinephrine reuptake inhibitors' (SNRIs). Other treatments may also be used, for example a type of physiotherapy known as vestibular rehabilitation, or talking therapies.

**What did we want to find out?**

We wanted to find out:

‐ whether there was evidence that medication (SSRIs and SNRIs) helps to improve the symptoms of PPPD;

‐ whether these treatments might cause any harm.

**What did we do?**

We searched for studies that compared SSRIs and SNRIs to either no treatment or placebo (dummy) treatment.

**What did we find?**

We did not find any studies that assessed the use of these medicines for PPPD. Therefore, we do not know if SSRIs and SNRIs are effective for the treatment of PPPD, or whether they might cause any harm.

**What are the limitations of the evidence?**

Further research is needed to identify whether these medications are useful for the treatment of PPPD.

**How up‐to‐date is this evidence?**

The evidence is up‐to‐date to November 2022.

## Summary of findings

**Summary of findings 1:** SSRIs and SNRIs compared to placebo or no treatment for PPPD

**SSRIs and SNRIs compared to placebo or no treatment for PPPD**						
**Patient or population:** adults with PPPD **Setting:** outpatient **Intervention:** SSRIs and SNRIs **Comparison:** placebo or no treatment						
**Outcomes**	Anticipated absolute effects^*^ (95% CI)		**Relative effect (95% CI)**	**№ of participants (studies)**	**Certainty of the evidence (GRADE)**	**Comments**
	**Risk with placebo**	**Risk with SSRIs and SNRIs**				
Improvement in vertigo at 6 to ≤ 12 months	No studies assessed this outcome.					
Change in vertigo at 6 to ≤ 12 months	No studies assessed this outcome.					
Serious adverse events	No studies assessed this outcome.					

**CI:** confidence interval; **PPPD:** persistent postural‐perceptual dizziness; **SNRI:** serotonin and norepinephrine reuptake inhibitor; **SSRI:** selective serotonin reuptake inhibitor

## Background

### Description of the condition

Persistent postural‐perceptual dizziness (PPPD) is a chronic balance disorder that is characterised by subjective unsteadiness or dizziness that is worse when standing up, moving around and in the presence of rich visual stimulation. Although the disorder was only defined in 2017, descriptions of individuals with the characteristic symptoms have been reported in the medical literature for many years (Staab 2017). The term itself has been used since at least 2013 (Staab 2020). In the past, individuals with these, or very similar, symptoms have been diagnosed with a variety of disorders, such as phobic postural vertigo, space‐motion discomfort, visual vertigo or chronic subjective dizziness (Staab 2017). PPPD includes the core features of many of these disorders. 

Criteria for diagnosis were established by expert consensus in 2017 and are based on symptoms alone. The presence of each of the following five features is required to make the diagnosis:

- dizziness, unsteadiness or non‐spinning vertigo, present on most days for at least three months;
- the symptoms are exacerbated by an upright posture, motion or exposure to complex visual stimuli;
- the disorder is triggered by an episode of unsteadiness, dizziness or vertigo ‐ caused by another balance disorder, a neurological or medical disorder, or psychological distress;
- symptoms must cause considerable distress to the sufferer;
- the symptoms should not be better accounted for by an alternative diagnosis.

As the diagnostic criteria were only recently established, accurate estimates of the prevalence and incidence of this newly characterised disorder are not yet available. However, a significant number of individuals with chronic balance problems, previously diagnosed with other conditions, may now be included within this diagnostic category. 

The pathophysiological processes underlying PPPD are incompletely understood, although a model has been proposed to explain the likely mechanism (Staab 2020). This suggests that temporary changes in balance function caused by a specific event (such as an acute balance disorder, medical or psychological disturbance) become chronic, despite the resolution of the initial insult. Balance function appears to become more dependent on visual input, and individuals may be hypervigilant with regard to their own movement and balance. PPPD may reflect a maladaptation to an acute vestibular insult.

The impact of PPPD on the individual may be considerable, due to the chronic and persistent nature of the condition, and the consequences it has for day‐to‐day activities and quality of life. A small qualitative study recently identified three themes describing the impact of this disorder on individuals (Sezier 2019). These were a perception that their symptoms were not viewed as part of a valid or credible disorder, a change in their perceived self‐identity since their symptoms started, and challenges in coping with the symptoms and changes in their lives. 

### Description of the intervention

In the absence of a good understanding of the pathophysiological mechanisms underlying PPPD, it is difficult to identify potential therapies based on any *specific* mechanisms. However, a variety of drugs and non‐drug interventions have been used in people with balance disorders characterised by similar symptoms, and these are therefore considered possible therapeutic options in people with PPPD. 

A small number of pharmacological interventions have been used for the treatment of PPPD (Popkirov 2018; Staab 2020). Selective serotonin reuptake inhibitors (SSRIs) and serotonin‐norepinephrine reuptake inhibitors (SNRIs) are, at present, the most commonly used medications for this disorder. Both are more widely used for depression and anxiety disorders. They are administered as oral tablets, usually titrating the dose up from a low starting level to reach the therapeutic range. Both classes of drug have a slow onset of action and, for depressive disorders, it may take several weeks before any benefit is seen. 

### How the intervention might work

Given the uncertainty in the pathogenesis of PPPD, at present no clear mechanism of action has been established for SSRIs and SNRIs. Possible modes of action may include altering hyperexcitability, or improving psychological symptoms (such as anxiety) that are present in many people with PPPD, or they may have direct effects on the widespread balance network in the brain. 

SSRIs act by preventing serotonin reabsorption by neurons, increasing serotonin levels in the brain. SNRIs have a similar mode of action, but inhibit the reuptake of both serotonin and norepinephrine. Serotonin receptors are found in the vestibular pathways within the brain (Balaban 2002), and there may consequently be direct actions of SSRIs and SNRIs on balance. 

It is also recognised that many individuals with chronic dizziness have associated symptoms, such as anxiety, mood disturbance and panic attacks. The use of SSRIs and SNRIs in PPPD may help to alleviate some of these symptoms, with resulting improvement in quality of life.

### Why it is important to do this review

Balance disorders can be difficult to diagnose and treat. There are few specific diagnostic tests, a variety of related disorders, and a limited number of interventions that are known to be effective. To determine which topics within this area should be addressed with new or updated systematic reviews, we conducted a scoping and prioritisation process, involving stakeholders (https://ent.cochrane.org/balance-disorders-ent). PPPD was ranked as one of the highest priority topics during this process (along with vestibular migraine and Ménière's disease). 

The impact on quality of life, and the absence of national or international management guidelines to recommend treatment strategies, make it important to review the evidence available to manage this condition. At present, there is no guidance available for healthcare professionals and patients to identify the possible benefits or harms of different treatment options. In this review, we aim to summarise the current evidence for pharmacological treatments for this condition; non‐pharmacological therapies are addressed in another review (Webster 2022a). 

## Objectives

To assess the benefits and harms of pharmacological interventions for persistent postural‐perceptual dizziness (PPPD). 

## Methods

### Criteria for considering studies for this review

#### Types of studies

We planned to include randomised controlled trials (RCTs) and quasi‐randomised trials (where trials were designed as RCTs, but the sequence generation for allocation of treatment used methods such as alternate allocation, birth dates etc). We also intended to include cross‐over trials and cluster‐randomised trials, if we could correctly account for the correlation in the data. 

For studies to obtain accurate estimates of the effect of different interventions, we considered that follow‐up of participants should be for at least three months, as the medications may take some time to take effect, and this is a chronic illness, where short‐term follow‐up may not accurately represent the longer‐term outcome for patients. Studies that followed up participants for less than three months were excluded from the review.

#### Types of participants

We included studies that recruited adult participants (aged 18 years or older) with a diagnosis of PPPD, according to the Bárány Society criteria (see Appendix 1). We excluded from the review studies that used alternative definitions of functional dizziness syndromes, such as chronic subjective dizziness (CSD), visual vertigo, space‐motion discomfort or phobic postural vertigo. Although we recognise that the symptoms of PPPD overlap considerably with some features of these disorders, we aimed to focus the results of the review so that they are directly relevant to those who are diagnosed with this (recently characterised) condition. 

#### Types of interventions

We included the following interventions:

- selective serotonin reuptake inhibitors (SSRIs);
- serotonin and norepinephrine reuptake inhibitors (SNRIs).

The main comparison was:

- SSRIs and SNRIs versus placebo/no treatment.

#### Types of outcome measures

We planned to assess outcomes at the following time points:

- 3 to < 6 months;
- 6 to ≤ 12 months;
- > 12 months.

The exception was for adverse event data, when we planned to use the longest time period of follow‐up. 

We searched the COMET database for existing core outcome sets of relevance to PPPD and vertigo, but were unable to find any published core outcome sets. We therefore conducted a survey of individuals with experience of (or an interest in) balance disorders to help identify outcomes that should be prioritised. This online survey was conducted with the support of the Ménière's Society and the Migraine Trust, and included 324 participants, who provided information regarding priority outcomes. The review author team used the results of this survey to inform the choice of outcome measures in this review. 

We planned to analyse the following outcomes in the review, but we did not use them as a basis for including or excluding studies.

##### Primary outcomes

- Improvement in vestibular symptoms
  - Measured as a dichotomous outcome (improved/not improved), according to self‐report, or according to a change of a specified score (as described by the study authors) on a rating scale.
- Change in vestibular symptoms
  - Measured as a continuous outcome, to identify the extent of change in vestibular symptoms.
- Serious adverse events
  - Including any event that caused death, was life‐threatening, required hospitalisation, resulted in disability or permanent damage, or in congenital abnormality. Measured as the number of participants who experienced at least one serious adverse event during the follow‐up period.

##### Secondary outcomes

- Disease‐specific health‐related quality of life
  - Measured with  
    - the Dizziness Handicap Inventory (DHI, Jacobsen 1990);
    - the DHI short form (Tesio 1999); or
    - the DHI screening tool (Jacobsen 1998).
- Generic health‐related quality of life
  - Measured with a validated measurement tool that assesses global health‐related quality of life, such as the EQ‐5D‐3L (EuroQol 1990), EQ‐5D‐5L (Herdman 2011) or SF‐36 (Ware 1992).
- Other adverse effects
  - Measured as the number of participants who experienced at least one episode of the specified adverse events during the follow‐up period, including the following specified adverse effects:
    - headache;
    - gastrointestinal disturbance;
    - sleep disturbance (e.g. somnolence or insomnia);
    - psychological disturbance (e.g. anxiety, depression, agitation);
    - cardiovascular disturbance (e.g. postural lightheadedness, palpitations);
    - sexual dysfunction.

### Search methods for identification of studies

The Cochrane ENT Information Specialist conducted systematic searches for randomised controlled trials and controlled clinical trials. There were no language or publication status restrictions. We only included studies that used the definition of PPPD that was defined in 2017 (Staab 2017), and first proposed in 2013 (Staab 2020). Therefore, we restricted some of the broader search terms to a year of publication from 2010 onwards. The date of the search was 21 November 2022.

#### Electronic searches

The Information Specialist searched:

- the Cochrane ENT Trials Register (search via the Cochrane Register of Studies to 21 November 2022);
- the Cochrane Central Register of Controlled Trials (CENTRAL) (search via the Cochrane Register of Studies to 21 November 2022);
- Ovid MEDLINE(R) Epub Ahead of Print, In‐Process & Other Non‐Indexed Citations, Ovid MEDLINE(R) Daily and Ovid MEDLINE(R) (1946 to 21 November 2022);
- Ovid Embase (1974 to 21 November 2022);
- Web of Knowledge, Web of Science (1945 to 21 November 2022);
- ClinicalTrials.gov, www.clinicaltrials.gov (searched to 21 November 2022);
- World Health Organization (WHO) International Clinical Trials Registry Platform (ICTRP), https://trialsearch.who.int (searched to 21 November 2022).

The Information Specialist modelled subject strategies for databases on the search strategy designed for CENTRAL, Ovid MEDLINE and Ovid Embase. Where appropriate, they were combined with subject strategy adaptations of the highly sensitive search strategy designed by Cochrane for identifying randomised controlled trials and controlled clinical trials (as described in the Technical Supplement to Chapter 4 of the *Cochrane Handbook for Systematic Reviews of Interventions* version 6.1) (Lefebvre 2021).

#### Searching other resources

We scanned the reference lists of identified publications for additional trials and contacted trial authors where necessary. In addition, the Information Specialist searched Ovid MEDLINE to retrieve existing systematic reviews relevant to this systematic review, so that we could scan their reference lists for additional trials. The Information Specialist also ran non‐systematic searches of Google Scholar to retrieve grey literature and other sources of potential trials.

We did not perform a separate search for adverse effects. We considered adverse effects described in included studies only.

### Data collection and analysis

#### Selection of studies

At least two review authors or co‐workers (of KG, TK, LS, KW) independently screened the titles and abstracts using Covidence (https://www.covidence.org), to identify studies that may be relevant for this review. Any discrepancies were resolved by consensus, or by retrieving the full text of the study for further assessment. 

The full text of any study that appeared potentially relevant was obtained and again checked by two authors or co‐workers (of KG, TK, LS, KW) independently to determine whether it met the inclusion criteria for the review. Any differences were resolved by discussion and consensus, or through recourse to a third author if necessary. Studies that were retrieved in full text but subsequently deemed to be inappropriate for the review (according to the inclusion/exclusion criteria) are listed as excluded studies, according to the main reason for exclusion. We recorded the study selection process in sufficient detail to complete a PRISMA flow diagram (Figure 1) and the Characteristics of included studies table. 

**1:**
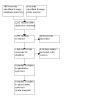


##### Screening eligible studies for trustworthiness

We planned to assess all studies meeting our inclusion criteria for trustworthiness using a screening tool developed by Cochrane Pregnancy and Childbirth. This tool includes specified criteria to identify studies that are considered sufficiently trustworthy to be included in the review (see Appendix 2). The process is outlined in Figure 2.

**2:**
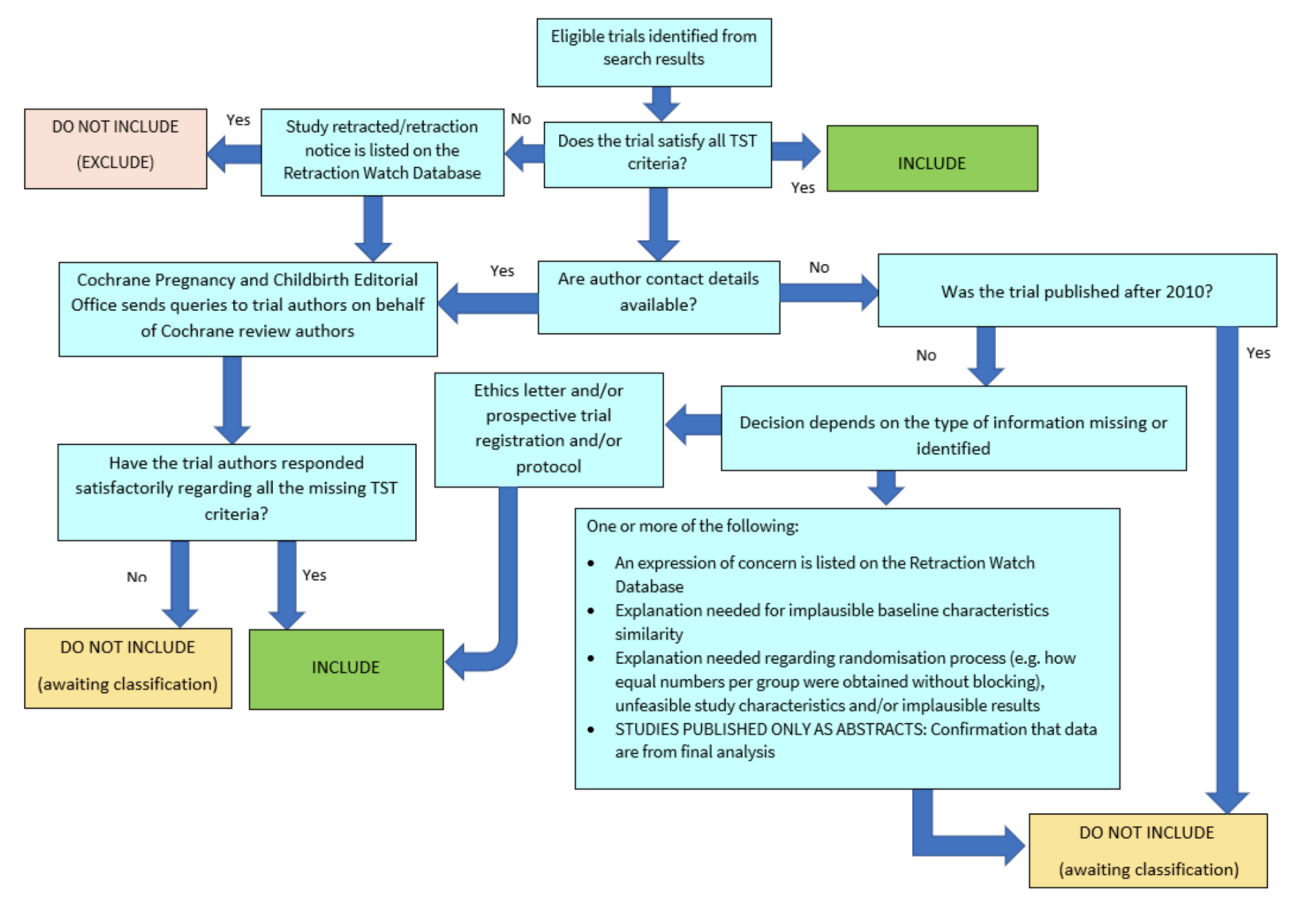
The Cochrane Pregnancy and Childbirth Trustworthiness Screening Tool

#### Data extraction and management

We planned that at least two review authors would independently extract outcome data from each study using a standardised data collection form, to include key characteristics of the studies. However, as no studies were identified for inclusion, this was not performed. 

#### Assessment of risk of bias in included studies

We planned that two authors would undertake assessment of the risk of bias of any included studies independently, as guided by the *Cochrane Handbook for Systematic Reviews of Interventions* (Handbook 2011). 

#### Measures of treatment effect

We planned to summarise the effects of dichotomous outcomes (e.g. serious adverse effects) as risk ratios (RR) with 95% confidence intervals (CIs). For continuous outcomes, we would have expressed treatment effects as a mean difference (MD) with standard deviation (SD) or as a standardised mean difference (SMD) if different scales had been used to measure the same outcome.

#### Unit of analysis issues

We did not encounter any unit of analysis issues, as we did not include any studies in this review. 

#### Dealing with missing data

We did not identify any studies suitable for inclusion in this review, therefore we did not need to contact authors regarding missing data. 

#### Assessment of heterogeneity

As no studies were included, we did not need to make an assessment of heterogeneity.

#### Assessment of reporting biases

See the protocol for more information on how we had planned to assess reporting bias (Webster 2022b). 

#### Data synthesis

See the protocol for more information on how we had planned to synthesise the data (Webster 2022b). 

#### Subgroup analysis and investigation of heterogeneity

If statistical heterogeneity was identified, we planned to assess this considering the following subgroups:

- Different types of medication, within a specific class.
- Different doses/frequency of administration.
- Use of concomitant treatment.

#### Sensitivity analysis

See the protocol for more information on the sensitivity analyses that we had planned to conduct (Webster 2022b). 

#### Summary of findings and assessment of the certainty of the evidence

We planned to use the GRADE approach to rate the overall certainty of evidence using GRADEpro GDT (https://gradepro.org/) and the guidance in Chapter 14 of the *Cochrane Handbook for Systematic Reviews of Interventions* (Handbook 2021).

We have prepared a summary of findings table for the main comparison:

- SSRIs and SNRIs versus placebo/no treatment.

We included the primary outcomes in the summary of findings table and prioritised outcomes at the time point 6 to ≤ 12 months.

## Results

### Description of studies

#### Results of the search

The searches in November 2022 retrieved a total of 1803 records. This reduced to 1233 after the removal of duplicates. We screened the titles and abstracts of these 1233 records. We discarded 1232 records and assessed one full‐text record, which was linked to one study. 

We excluded one study (one record) with reasons recorded in the review (see Excluded studies). We did not identify any studies that were relevant for inclusion in this review. We also did not identify any ongoing studies for this review. 

A flow chart of study retrieval and selection is provided in Figure 1.

#### Included studies

No studies were included in the review.

#### Excluded studies

We excluded one study from this review (Basta 2017). This was a trial of cinnarizine and dimenhydrinate for people undergoing vestibular rehabilitation. However, participants in this trial did not have PPPD ‐ they were diagnosed with a variety of other balance disorders. 

### Risk of bias in included studies

No studies were included in the review.

### Effects of interventions

See: Table 1

No studies were included in the review.

## Discussion

### Summary of main results

No studies were included in this review.

### Overall completeness and applicability of evidence

At present, there is no evidence from placebo‐controlled randomised controlled trials (RCTs) regarding the efficacy and harms of selective serotonin reuptake inhibitors (SSRIs) or serotonin and norepinephrine reuptake inhibitors (SNRIs) for persistent postural‐perceptual dizziness (PPPD).  

### Quality of the evidence

We planned to use GRADE to assess the certainty of the evidence for each of our outcomes. However, as we identified no studies for inclusion in this review we are not able to comment on the certainty of the evidence. 

### Potential biases in the review process

We adhered to our protocol during the review process. However, some decisions about relevant studies to include in this review may be subject to some debate. Firstly, we only included studies that compared SSRIs or SNRIs to a placebo or no treatment control group. This was because we did not consider there to be a 'gold standard' treatment available for PPPD. Therefore, to establish whether these interventions may be effective, we needed to compare them to no treatment, or ‐ ideally ‐ a placebo. 

Secondly, we did not include any studies that followed up participants for less than three months. This was because we considered that the efficacy of an intervention in a chronic condition, such as PPPD, could not be judged with very short follow‐up. Even if an intervention is effective at one month or two months, it does not mean that there will be persisting efficacy in the long term. However, despite this restriction, we did not identify any studies that were excluded on the basis of this criterion. Therefore, we think it unlikely that the review would be different if studies with shorter follow‐up had been included. 

Finally, in keeping with our protocol, we only included studies where participants had received a diagnosis of PPPD using the Bárány society criteria. This may have led to the omission of studies that predated these criteria and recruited participants with different ‐ but related ‐ diagnoses, such as chronic subjective dizziness or phobic postural vertigo. This could be regarded as a bias in the review process. Nonetheless, we considered it vital to focus the review on those people with a definitive diagnosis of PPPD, to assess the current evidence for this specific condition. 

### Agreements and disagreements with other studies or reviews

There are few published reviews that consider the efficacy and harms of interventions for PPPD. We did not identify any other systematic reviews that addressed this question. However, we note the findings of a recent narrative review on this topic, which also highlights the lack of RCTs in this field (Staab 2020). Results from a single RCT were included in this article (Yu 2018). However, this considered a non‐pharmacological intervention (cognitive behavioural therapy) as an adjunct to sertraline treatment (an SSRI) for PPPD.

## Authors' conclusions

**Implications for practice**

At present there is no evidence regarding the efficacy or potential harms of the use of selective serotonin reuptake inhibitors (SSRIs) or serotonin and norepinephrine reuptake inhibitors (SNRIs) for persistent postural‐perceptual dizziness (PPPD). People with this condition, and healthcare professionals who work with them, should be aware of this uncertainty when deciding on interventions to use for their condition. 

It should be noted that SSRIs and SNRIs are widely used for other conditions and have been associated with well‐recognised side effects, including psychological disturbance and gastrointestinal symptoms. The potential for these adverse effects should be considered when weighing up the risks and benefits of treatment. 

**Implications for research**

Further work is needed in this area to identify whether any interventions are effective in the treatment of PPPD, and whether they are associated with any harms. The following conclusions relate to the evidence identified in this review, and a companion review on non‐pharmacological interventions for PPPD:

- Authors of future studies should ensure that the agreed diagnostic criteria for PPPD are used to identify study participants (Appendix 1). 
- There is currently no evidence from randomised controlled trials to support a 'gold standard' treatment for PPPD, although some interventions are in widespread use. Placebo‐controlled trials are therefore vital to identify the potential efficacy of interventions for PPPD. Comparison with other interventions (of unknown efficacy) does not allow firm conclusions to be drawn. 
- PPPD is a chronic condition, with symptoms that can recur over months or years. This needs to be considered when designing clinical trials in this area, and determining the appropriate duration of follow‐up. A number of studies were found in the literature that conducted short‐term follow‐up and assessed outcomes at four or eight weeks. We considered that it is not possible to draw conclusions about the efficacy of these interventions with such short follow‐up. We would advocate that authors of future studies plan for a longer duration of follow‐up to establish whether interventions have benefit for the long‐term symptoms of this disorder. 
- As with other balance disorders, there should be agreement about which outcomes to measure in studies of PPPD, and how to measure them. There should also be agreement about what size of a difference in symptoms would be meaningful and important to people with this condition. This can only be achieved through collaboration between people with PPPD, healthcare professionals and researchers, and development of a core outcome set would be advantageous. 

## History

Protocol first published: Issue 3, 2022

## Appendices

### Appendix 1. Diagnostic criteria for PPPD

The Bárány Society criteria for PPPD (Staab 2017):

1. One or more symptoms of dizziness, unsteadiness or non‐spinning vertigo, present on most days for three months or more:
  - symptoms are persistent, but wax and wane;
  - symptoms tend to increase as the day progresses, but may not be active throughout the entire day;
  - momentary flares may occur spontaneously or with sudden movements.
2. Symptoms are present without specific provocation, but are exacerbated by:
  - upright posture;
  - active or passive motion without regard to direction or position; and
  - exposure to moving visual stimuli or complex visual patterns, although these three features may not be equally provocative.
3. The disorder usually begins shortly after an event that causes acute vestibular symptoms or problems with balance, though less commonly it develops slowly. Precipitating events include acute, episodic or chronic vestibular syndromes, other neurologic or medical illness, and psychological distress.
  - When triggered by an acute or episodic precipitant, symptoms typically settle into the pattern of criterion 1 as the precipitant resolves, but may occur intermittently at first, and then consolidate into a persistent course.
  - When triggered by a chronic precipitant, symptoms may develop slowly and worsen gradually.
4. Symptoms cause significant distress or functional impairment.
5. Symptoms are not better attributed to another disease or disorder.

### Appendix 2. Trustworthiness Screening Tool

This screening tool has been developed by Cochrane Pregnancy and Childbirth. It includes a set of predefined criteria to select studies that, based on available information, are deemed to be sufficiently trustworthy to be included in the analysis. These criteria are:

**Research governance**

- Are there any retraction notices or expressions of concern listed on the Retraction Watch Database relating to this study?
- Was the study prospectively registered (for those studies published after 2010)? If not, was there a plausible reason?
- When requested, did the trial authors provide/share the protocol and/or ethics approval letter?
- Did the trial authors engage in communication with the Cochrane Review authors within the agreed timelines?
- Did the trial authors provide IPD data upon request? If not, was there a plausible reason?

**Baseline characteristics**

- Is the study free from characteristics of the study participants that appear too similar (e.g. distribution of the mean (SD) excessively narrow or excessively wide, as noted by Carlisle 2017)?

**Feasibility**

- Is the study free from characteristics that could be implausible? (e.g. large numbers of women with a rare condition (such as severe cholestasis in pregnancy) recruited within 12 months);
- In cases with (close to) zero losses to follow‐up, is there a plausible explanation?

**Results**

- Is the study free from results that could be implausible? (e.g. massive risk reduction for main outcomes with small sample size)?
- Do the numbers randomised to each group suggest that adequate randomisation methods were used (e.g. is the study free from issues such as unexpectedly even numbers of women ‘randomised’ including a mismatch between the numbers and the methods, if the authors say ‘no blocking was used’ but still end up with equal numbers, or if the authors say they used ‘blocks of 4’ but the final numbers differ by 6)?

Studies assessed as being potentially ‘high risk’ will be not be included in the review. Where a study is classified as ‘high risk’ for one or more of the above criteria we will attempt to contact the study authors to address any possible lack of information/concerns. If adequate information remains unavailable, the study will remain in ‘awaiting classification’ and the reasons and communications with the author (or lack of) described in detail.

The process is described in full in Figure 2.

### Appendix 3. Search strategies

**Table:**

**CENTRAL (CRS)**	**MEDLINE (Ovid)**	**Embase (Ovid)**
1 MESH DESCRIPTOR Dizziness EXPLODE ALL AND CENTRAL:TARGET 809 2 MESH DESCRIPTOR Vertigo AND CENTRAL:TARGET 509 3 MESH DESCRIPTOR Postural Balance AND CENTRAL:TARGET 0 4 MESH DESCRIPTOR Vestibular Diseases AND CENTRAL:TARGET 0 5 MESH DESCRIPTOR Motion perception EXPLODE ALL AND CENTRAL:TARGET 299 6 #1 OR #2 OR #3 OR #4 OR #5 1515 7 MESH DESCRIPTOR Chronic Disease EXPLODE ALL AND CENTRAL:TARGET 13660 8 (chronic or persistent or "long term" or longstanding):AB,TI,TO AND CENTRAL:TARGET 245806 9 #7 OR #8 247437 10 #6 AND #9 190 11 (chronic or persistent OR persisting or "long term" or longstanding) adj6 (dizziness OR dizzy OR vertigo OR vestibular):AB,TI,TO AND CENTRAL:TARGET 294 12 #10 OR #11 405 13 >2009:YR AND CENTRAL:TARGET 1020392 14 #12 AND #13 269 15 MESH DESCRIPTOR Perceptual Disorders EXPLODE ALL AND INSEGMENT 175 16 #6 AND #15 3 17 (persistent adj3 (perceptual or postural) adj3 (dizziness or vertigo)):AB,EH,KW,KY,MC,MH,TI,TO AND CENTRAL:TARGET 12 18 (PPPD AND (dizziness or vertigo or vestibular)):AB,EH,KW,KY,MC,MH,TI,TO AND CENTRAL:TARGET 11 19 #14 OR #16 OR #17 OR #18 273	MEDLINE (Ovid MEDLINE® Epub Ahead of Print, In‐Process & Other Non‐Indexed Citations, Ovid MEDLINE® Daily and Ovid MEDLINE®) 1946 to present 1 exp Dizziness/ 5763 2 Vertigo/ 10478 3 Postural Balance/ 25602 4 Vestibular Diseases/ 4511 5 exp Motion perception/ 16966 6 1 or 2 or 3 or 4 or 5 59217 7 exp Chronic Disease/ 271574 8 6 and 7 847 9 ((persistent or chronic or persisting or "long term" or longstanding) adj6 (dizziness or dizzy or vertigo or vestibular)).ab,ti. 1765 10 8 or 9 2439 11 (201* or 202*).yr. 13228363 12 10 and 11 1383 13 Perceptual Disorders/ 6739 14 13 and 10 11 15 (persistent adj3 (perceptual or postural) adj3 (dizziness or vertigo)).ab,ti. 102 16 (PPPD and (dizziness or vertigo or vestibular)).ab,ti. 75 17 12 or 14 or 15 or 16 1389 18 randomized controlled trial.pt. 545668 19 controlled clinical trial.pt. 94445 20 randomized.ab. 536242 21 placebo.ab. 222027 22 drug therapy.fs. 2382579 23 randomly.ab. 367234 24 trial.ab. 570881 25 groups.ab. 2255489 26 18 or 19 or 20 or 21 or 22 or 23 or 24 or 25 5137324 27 exp animals/ not humans.sh. 4894687 28 26 not 27 4469024 29 17 and 28 494	Embase 1974 to present 1 exp dizziness/ 84247 2 vertigo/ 47669 3 body equilibrium/ 20518 4 vestibular disorder/ 9404 5 exp movement perception/ 13785 6 1 or 2 or 3 or 4 or 5 164913 7 exp chronic disease/ 190351 8 6 and 7 1397 9 ((persistent or chronic or persisting or longstanding or "long term") adj6 (dizziness or dizzy or vertigo or vestibular)).ab,ti. 2406 10 8 or 9 3628 11 (201* or 202*).yr. 17776219 12 10 and 11 2234 13 exp perception disorder/ 35625 14 10 and 13 45 15 (persistent adj3 (perceptual or postural) adj3 (dizziness or vertigo)).ab,ti. 132 16 (PPPD and (dizziness or vertigo or vestibular)).ab,ti. 101 17 12 or 14 or 15 or 16 2248 18 Randomized controlled trial/ 678551 19 Controlled clinical study/ 464183 20 Random$.ti,ab. 1711730 21 randomization/ 91931 22 intermethod comparison/ 275752 23 placebo.ti,ab. 330279 24 (compare or compared or comparison).ti. 547713 25 ((evaluated or evaluate or evaluating or assessed or assess) and (compare or compared or comparing or comparison)).ab. 2377142 26 (open adj label).ti,ab. 91422 27 ((double or single or doubly or singly) adj (blind or blinded or blindly)).ti,ab. 249007 28 double blind procedure/ 188384 29 parallel group$1.ti,ab. 28187 30 (crossover or cross over).ti,ab. 112909 31 ((assign$ or match or matched or allocation) adj5 (alternate or group$1 or intervention$1 or patient$1 or subject$1 or participant$1)).ti,ab. 364054 32 (assigned or allocated).ti,ab. 429177 33 (controlled adj7 (study or design or trial)).ti,ab. 389388 34 (volunteer or volunteers).ti,ab. 261134 35 human experiment/ 555942 36 trial.ti. 340283 37 18 or 19 or 20 or 21 or 22 or 23 or 24 or 25 or 26 or 27 or 28 or 29 or 30 or 31 or 32 or 33 or 34 or 35 or 36 5534662 38 (random$ adj sampl$ adj7 ("cross section$" or questionnaire$1 or survey$ or database$1)).ti,ab. 12284 39 comparative study/ or controlled study/ 9044969 40 randomi?ed controlled.ti,ab. 326332 41 randomly assigned.ti,ab. 144742 42 39 or 40 or 41 9230030 43 38 not 42 8726 44 Cross‐sectional study/ 437766 45 randomized controlled trial/ or controlled clinical study/ or controlled study/ 8500635 46 (randomi?ed controlled or control group$1).ti,ab. 993836 47 45 or 46 8866090 48 44 not 47 284171 49 (((case adj control$) and random$) not randomi?ed controlled).ti,ab. 18978 50 (Systematic review not (trial or study)).ti. 187767 51 (nonrandom$ not random$).ti,ab. 17282 52 "Random field$".ti,ab. 2590 53 (random cluster adj3 sampl$).ti,ab. 1386 54 (review.ab. and review.pt.) not trial.ti. 929038 55 "we searched".ab. 62552 56 review.ti. or review.pt. 3133557 57 55 and 56 38528 58 "update review".ab. 119 59 (databases adj4 searched).ab. 45639 60 (rat or rats or mouse or mice or swine or porcine or murine or sheep or lambs or pigs or piglets or rabbit or rabbits or cat or cats or dog or dogs or cattle or bovine or monkey or monkeys or trout or marmoset$1).ti. and animal experiment/ 1123962 61 43 or 48 or 49 or 50 or 51 or 52 or 53 or 54 or 57 or 58 or 59 1388815 62 37 not 61 5249339 63 17 and 62 551

## Characteristics of studies

### Characteristics of excluded studies [ordered by study ID]

**Table:**

Study	Reason for exclusion
Basta 2017	This RCT did not recruit participants with a diagnosis of PPPD. Instead, it included people with presbyvertigo, unilateral horizontal semicircular canal loss or unilateral saccular disorders.

PPPD: persistent postural‐perceptual dizziness RCT: randomised controlled trial

## Differences between protocol and review

We did not make any amendments to the protocol during the preparation of this review. 
